# Supplementary material for: Targeting Phospholipase D Pharmacologically Prevents Phagocytic Function Loss of Retinal Pigment Epithelium Cells Exposed to High Glucose Levels
Source: Int J Mol Sci. 2022 Oct 5;23(19):11823. doi: 10.3390/ijms231911823 (PMC9570224; doi:10.3390/ijms231911823)
Supplement: Supplementary file 1 [file ijms-23-11823-s001.zip › ijms-1936062-supplementary.pdf]

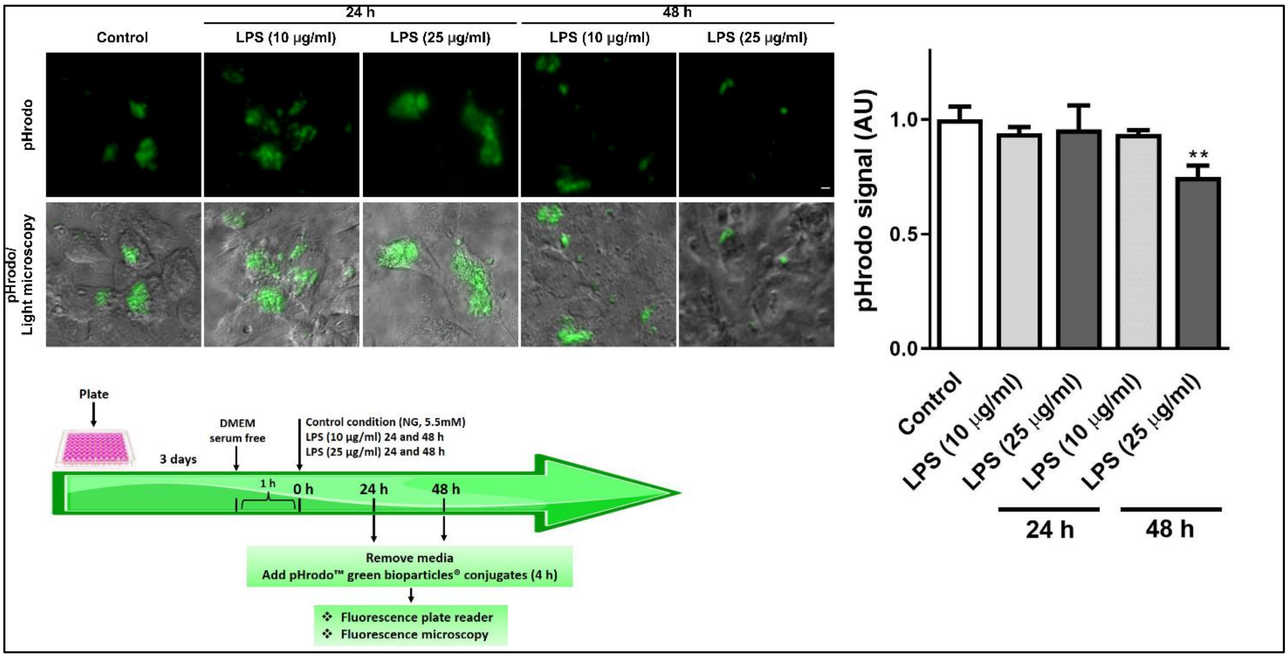


**Figure S1.** Non-specific phagocytosis in ARPE-19 cells exposed to lipopolysaccharide (LPS). ARPE-19 cells were seeded in 96-well plates and exposed to LPS (10 or 25 µg/ml) for 24 and 48 h. Non-specific phagocytosis was measured using pHrodo™ green bioparticles® conjugates. Representative fluorescence images are shown (Scale bar = 10 µm). Bar graph shows pHrodo fluorescence intensity expressed as arbitrary units (AU) with respect to control conditions. Asterisks (*) indicate significant differences with respect to control condition (** $p < 0.01$).

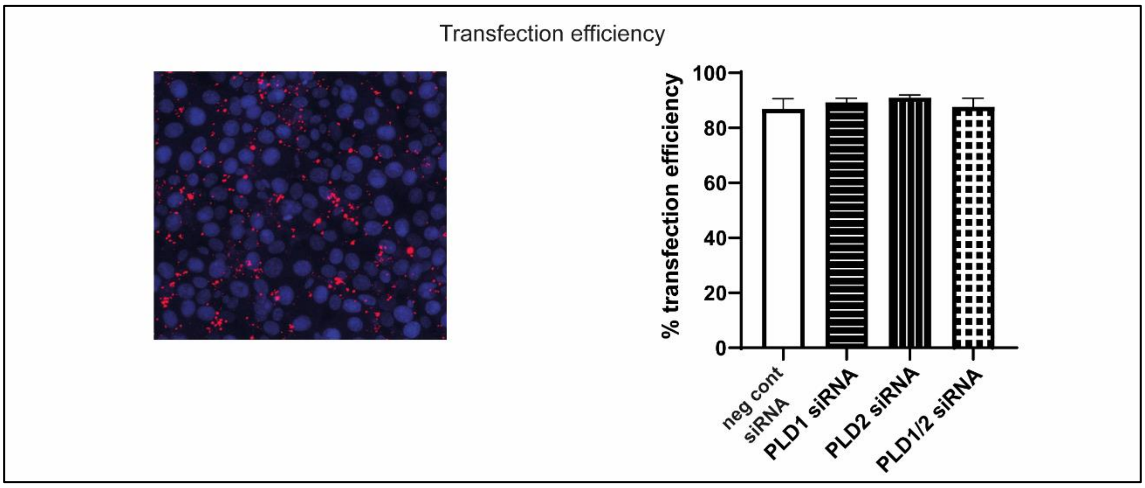


**Figure S2.** Transfection efficiency was obtained after quantification of AllStars Neg. siRNA AF 555 positive cells (red) and DAPI nuclear staining (blue) are shown. The latter was used as a transfection control in all the experimental samples. In all the cases a high transfection efficiency was achieved.

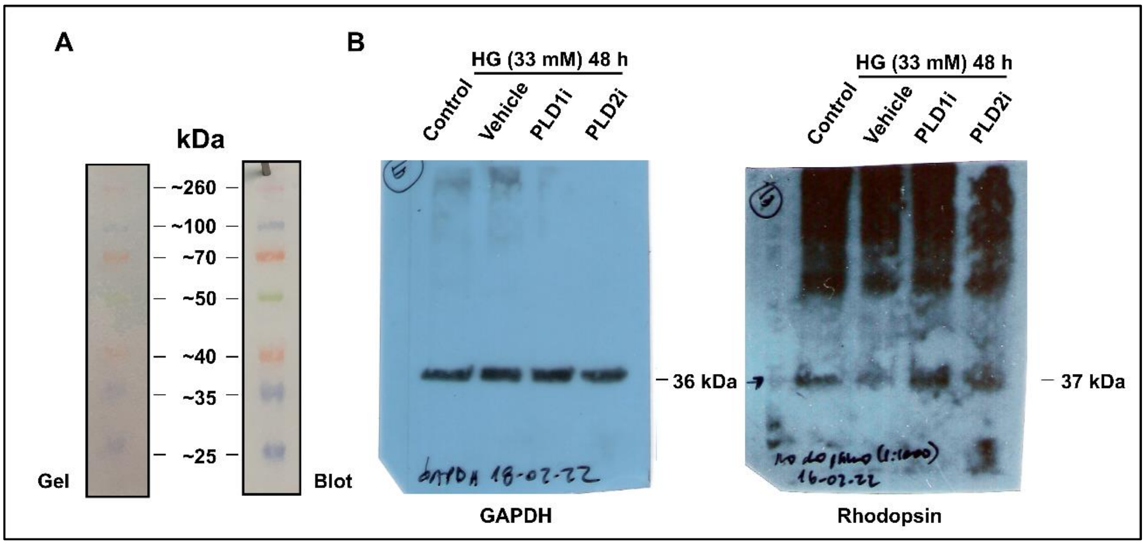


**Figure S3.** (**A**) Spectra multicolor broad range protein ladder. Molecular weights are in kDa. (**B**) Whole membranes of Rhodopsin and GAPDH western blotting in ARPE-19 cells. GAPDH was used as loading control.

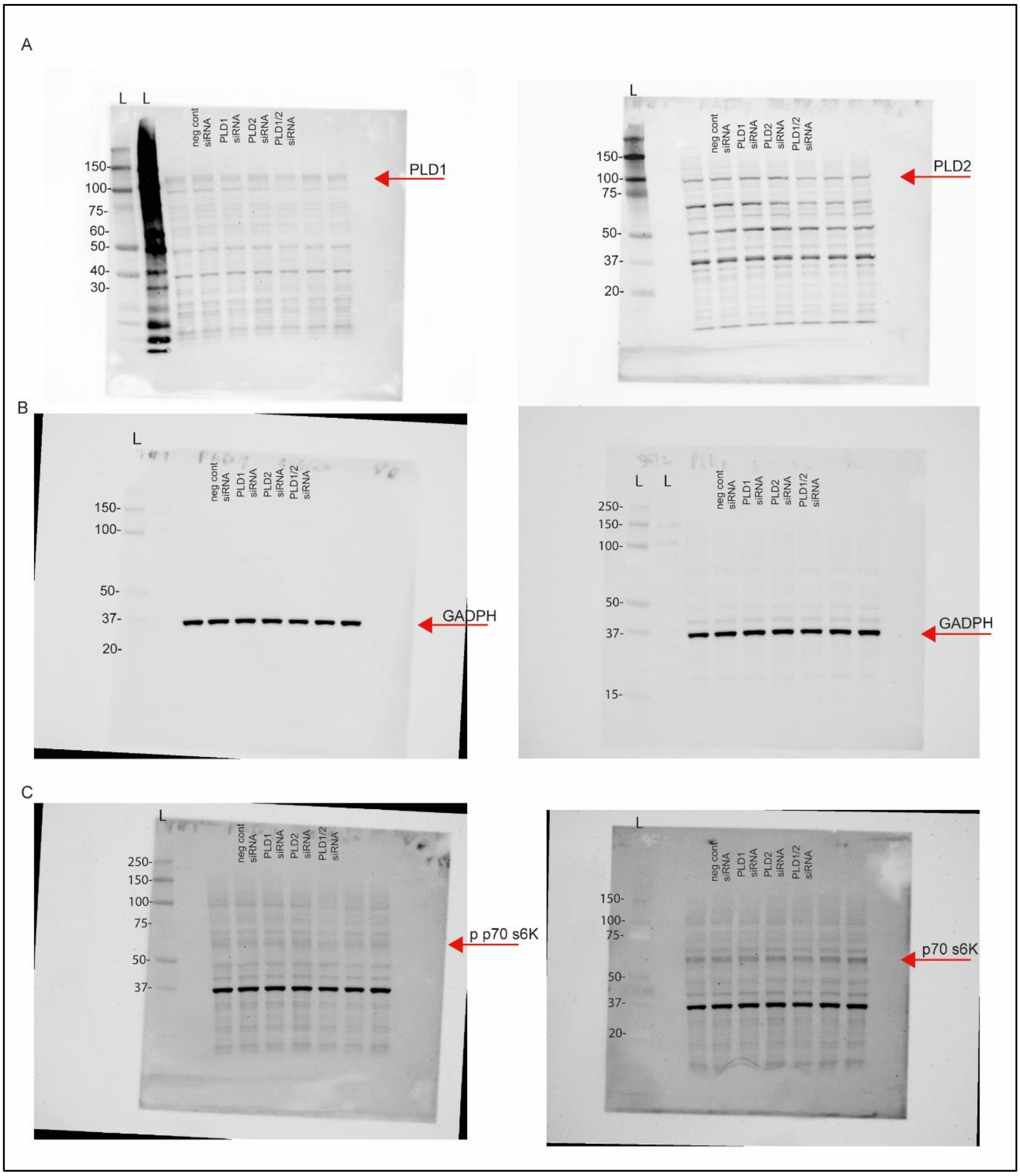


**Figure S4.** (**A**) Comparative protein expression of phospholipase D (PLD) 1 and 2 in PLD1 siRNA and PLD2 siRNA transfected ABC cells. (**B**) GAPDH was used as loading control. (**C**) Comparative protein expression of p p70 S6K and p70 S6K in PLD1 siRNA and PLD2 siRNA transfected ABC cells. L, protein ladder, molecular weights are in kDa.

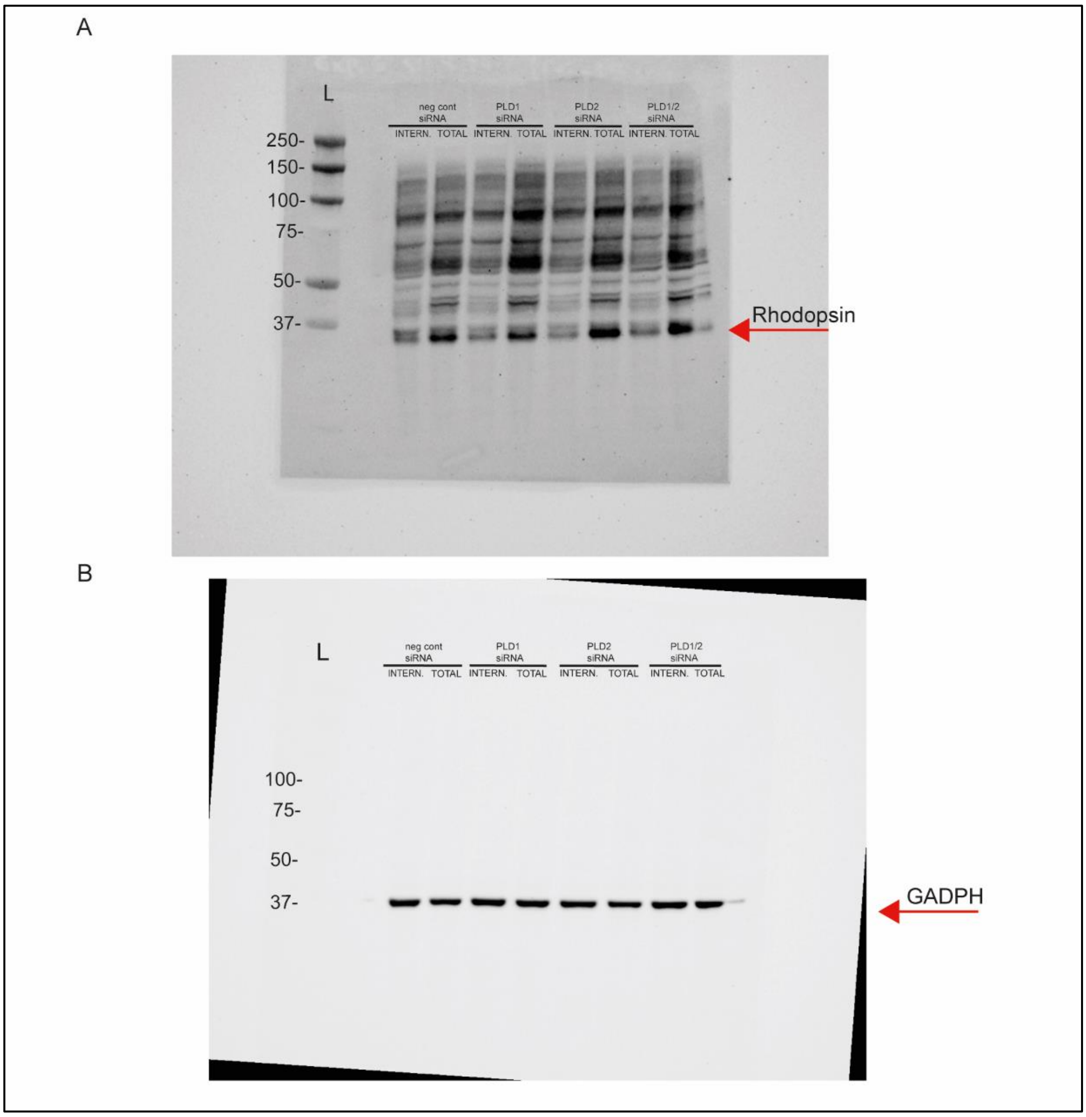


**Figure S5.** Whole membranes of Rhodopsin (**A**) and GAPDH (**B**) western blotting in PLD1 siRNA and PLD2 siRNA transfected ABC cells. GAPDH was used as loading control. L, protein ladder, molecular weights are in kDa.

**Table S1**

**ARPE-19 vs ABC cells**

| Name | Chromosome | Region | Max group mean | $Log_2$ fold change | Fold change | p-values | FDR p-values | ENSEMBL |
|---|---|---|---|---|---|---|---|---|
| PLD1 | 3 | Complement (171318195..171528740) | 3.497334274 | -0.251104267 | -1.190117706 | 0.04266702 | 0.254359878 | ENSG00000075651 |
| PLD2 | 17 | 4710391..4726729 | 5.831054375 | -0.443481455 | -1.359881985 | 0.00046259 | 0.004793733 | ENSG00000129219 |

**hRPE49 vs ABC cells**

| Name | Chromosome | Region | Max group mean | $Log_2$ fold change | Fold change | p-values | FDR p-values | ENSEMBL |
|---|---|---|---|---|---|---|---|---|
| PLD1 | 3 | Complement (171318195..171528740) | 3.497334 | -1.13387 | -2.19447 | 0 | 0 | ENSG00000075651 |
| PLD2 | 17 | 4710391..4726729 | 5.831054 | -0.60004 | -1.51576 | 3.62E-06 | 3.5E-05 | ENSG00000129219 |

ABC N=3
ARPE-19 N=3
hRPE49 N=2
